# Assessing the Association of Self-Esteem with Psychological Distress and Lifestyle in a Cohort of Tunisian Medical Students

**DOI:** 10.1192/j.eurpsy.2026.11315

**Published:** 2026-07-31

**Authors:** S. Ben Fredj, R. Ghammam, N. Zammit, A. Skhiri, O. Ben Saad, S. Rhimi, A. Chouchen, I. Harrabi

**Affiliations:** 1 Department of Preventive Medicine and Epidemiology, “LR19SP03”, University Hospital Farhat Hached; 2 Faculty of Medicine of Sousse, University of Sousse; 3 Department of Occupational Medicine, “LR19SP03”, University Hospital Farhat Hached , Sousse, Tunisia

## Abstract

**Introduction:**

Higher education exposes all students especially medical students to high levels of stress, which can lead to various mental disorders. Medical students face a unique confluence of challenges, including intense academic pressure, prolonged study hours, clinical responsibilities, and the emotional burden of patient care. Within this context, self-esteem —the individual’s overall subjective sense of personal worth and value —serves as a critical psychological resource.

**Objectives:**

This study aimed to assess the prevalence of self-esteem among students at the Faculty of Medicine of Sousse and its association with anxiety, depression, and lifestyle habits.

**Methods:**

We conducted a cross-sectional study among medical students at the Faculty of Medicine of Sousse (Tunisia), during the 2020-2021 academic year. Data was collected anonymously via an online questionnaire posted on each grade’s Facebook groups in December 2020. This questionnaire aimed to collect sociodemographic data, lifestyle habits, perception of body image, self-esteem using the Rosenberg Self-Esteem Scale (RSE), and anxiety and depression using the Hospital Anxiety and Depression Scale (HADS).

**Results:**

The study included 147 students, with an average age of 20.31±1.75 years, and female predominance (74.1%). The Rosenberg score revealed that 47.6% of participants had very low to low self-esteem, while 32% had high to very high self-esteem. according to the HAD scale, 22.4% of participants had anxiety symptoms and 25.2% had depression symptoms.

Students who do leisure activities had significantly higher self-esteem compared to students who don’t do these activities (p=0.017). Conversely, participants who reported alcohol consumption showed significantly lower self-esteem levels compared to non-consumers (p=0.026). Anxiety and depression were negatively correlated with self-esteem (B=**-**0.331, p<10^-3^; B=**-**0.504, p<10^-3^ respectively).

**Conclusions:**

The findings indicate a high prevalence of low self-esteem and psychological distress among medical students in Tunisia, with self-esteem being significantly correlated with lifestyle factors and mental health symptoms. These results underscore the necessity of implementing targeted support and wellness programs within medical education to promote psychological well-being and mitigate risk factors throughout students’ academic journeys.

**Disclosure of Interest:**

None Declared

